# The potential role of TRPV1 in pulmonary hypertension: Angel or demon?

**DOI:** 10.1080/19336950.2019.1631106

**Published:** 2019-06-17

**Authors:** Xin Zhang, Lifang Ye, Yu Huang, Xueyan Ding, Lihong Wang

**Affiliations:** a The Second Clinical Medical College, Zhejiang Chinese Medical University, Hangzhou, China; b Department of Cardiovascular Medicine, Zhejiang Provincial People's Hospital, People's Hospital of Hangzhou Medical College, Hangzhou, China

**Keywords:** Pulmonary hypertension, transient receptor potential vanilloid subfamily member 1, Ca^2+^, neuropeptides, inflammation

## Abstract

Pulmonary hypertension (PH) is a pathological state defined by increased pulmonary artery pressure, the pathogenesis of which is related to genetic mutations, intracellular calcium ([Ca^2+^]_i_), inflammation and proliferation. Transient receptor potential vanilloid subfamily member 1 (TRPV1) is a nonselective cation channel expressed in neural and nonneural cells, including pulmonary vessels and nerves. As a calcium channel, TRPV1 can make vessels contracted, and promote smooth muscle cells proliferation through calcium-dependent transcription factors. Activation of TRPV1 in sensory nerves can release neuropeptides, including calcitonin gene-related peptide (CGRP), substance P (SP), and somatostatin (SST), which can regulate inflammation via transcription factor NF-kB. Considering the increased level of [Ca^2+^]_i_ and inflammation in the pathogenesis of PH, our review summarizes the role of TRPV1 in PH with regard to [Ca^2+^]_i_, neuropeptides, and inflammation. In view of the limited research illustrating the relationship between TRPV1 and PH directly, our review also considers the role of TRPV1 in other types of vascular inflammation. Through this review, we hope to raise awareness about the function of TRPV1 in PH.

## Introduction

### Pulmonary hypertension

Pulmonary hypertension (PH) is defined as a mean pulmonary artery pressure (mPAP) surpassing 25 mmHg at resting conditions with right heart catheterization or a mPAP over 30 mmHg in a state of motion, although the latter criterion has been questioned due to its instability [1,2]. Pulmonary arterial hypertension (PAH) is a subtype of PH with the criteria of an end-expiratory pulmonary artery wedge pressure less than or equal to 15 mmHg and a pulmonary vascular resistance exceeding 3 Wood units [1]. These two pathological states have overlapping pathophysiology and pathogenesis in principle [3]. Therefore, the two terms will not be strictly segregated in this review. To date, there have been many experiments studying the pathogenesis of PAH, although its exact mechanism is still vague and needs further exploration. Based on previous studies, we conclude that the pathogenesis of PAH is closely associated with genetic mutations, such as bone morphogenetic protein receptor type 2 (BMPR2), and with cytosolic calcium and inflammation [3]. The increased intracellular calcium ([Ca^2+^]_i_) is involved in vessels shrinkage and proliferation, which is harmful to PAH via increased pulmonary artery resistance [4]. Another cause of PAH is inflammation, although the exact pathway remains unclear. There are four lines of evidence that can prove their relationship. First, specimens from PAH patients are accompanied by an accumulation of perivascular inflammatory cells, including lymphocytes, mast cells, monocytes and macrophages. Second, cytokines and chemokines in circulating blood are elevated in PAH patients. Third, certain inflammatory conditions such as connective tissue diseases are associated with an increased incidence of PAH. Finally, inflammation inhibitors, such as glucocorticoid, can alleviate experimentally induced PAH [3,5,6]. Although the exact formation mechanism of PAH is unclear, the outcome of PAH is scary, namely, right ventricle hypertrophy and, ultimately, life-threatening heart failure. Therefore, studying the mechanism of PAH and finding effective treatments are extremely urgent.

### Transient receptor potential vanilloid subfamily member 1

Transient receptor potential vanilloid subfamily member 1 (TRPV1), the vanilloid subtype of the transient receptor potential (TRP) family, is a nonselective cation channel that can allow passage of H^+^, Na^+^, Ca^2+^ and Mg^2+^ [7,8]. TRPV1 can be activated by heat, pain, stretch, acidic pH, capsaicin, vanilloids, resiniferatoxin, bradykinin, prostaglandins, adenosine triphosphate, and arachidonic acid metabolites, such as cannabinoids, N-arachidonoyl dopamine and N-oleoyldopamine; conversely, TRPV1 can be blocked by capsazepine, 5′-Iodoresiniferatoxin, AMG9810, SB366791, A-425619, ruthenium red, and 12-acetoxy-hawtriwaic acid lactone [8–10]. Apart from being widely distributed throughout the nervous system, TRPV1 can be found in various organs and tissues, including the heart, blood vessels, lungs, trachea, kidneys, skin, retinas, joints, intestines, brain, uterus, testes, salivary glands, and pancreas, and it plays an important role in pathogenic processes, such as atherosclerosis, ischemia/reperfusion (I/R) injury, myocardial fibrosis and remodeling, hypertension, asthma, arthritis, dermatitis, and diabetes [8,11–19]. At the cellular level, TRPV1 can be found in endothelial cells, vascular smooth muscle cells (VSMCs), platelets, mast cells, lymphocytes, macrophages, cardiac ventricles, the epicardial surface, sensory nerve fibers innervating smooth muscle, adventitia, myocardium and ventricular epicardial surface [8,10,17,20,21]. When activated by agonists, TRPV1 nerve terminals can induce the release of neuropeptides, such as calcitonin gene-related peptide (CGRP), substance P (SP), and somatostatin (SST), which are involved in inflammation and vascular events [15]. Nonneural TRPV1 is also associated with inflammation and shares a close relationship with Ca^2+^ [22,23].

Given the link between TRPV1 and PAH in Ca^2+^ and inflammation respects, a summary of their direct or indirect relationship is necessary. Our review will illustrate, at least in part, the influence of TRPV1 on the progression of PAH in terms of Ca^2+^, neuropeptides and inflammation. Additionally, due to the limited studies of the direct role of TRPV1 in PAH, especially in terms of inflammation, we extend the scope of objectives and aim to identify more effects of TRPV1 on the pulmonary artery by analyzing TRPV1 in other arteries. Hopefully, this information will contribute to the assessment of the role of TRPV1 in PAH and the exploration of new therapies.

## Ca^2+^ and TRPV1 in pulmonary artery smooth muscle cells

As is well known, TRPV1 is a nonselective cation channel that allows Ca^2+^ influx, so it is necessary to explore the Ca^2+^ signaling pathway to study the role of TRPV1 in the pathogenesis of PAH. Ca^2+^ is considered to be an important regulator of the resistance of vessels due to its vasoconstrictive, pro-proliferative and promigratory effects, and its concentration in pulmonary artery smooth muscle cells (PASMCs) is crucial for the formation of PAH, because a increased level of [Ca^2+^]_i_ is recorded by microspectrofluorimetric assay in PAH PASMCs [4,23–25]. There are three types of traditional calcium channels in PASMC membranes. One type includes voltage-independent calcium channels (VDC), which primarily function in excitable cells and muscle cells. Another type includes receptor-operated calcium channels (ROC) and store-operated calcium channels (SOC), which are voltage-independent and responsible for the regulation of vascular tone and the proliferation of vascular smooth muscle cells (VSMCs). As their names imply, they can be activated by the binding of membrane receptors to ligands and the depletion of calcium storage in the endoplasmic reticulum (ER), respectively. It is well accepted that the TRP family plays an important role in contributing to the formation of ROC and SOC in PASMCs [4,26]. The increased [Ca^2+^]_i_ in PAH PASMCs comes from the influx of calcium through the above three types of channels in cytomembranes, including TRPV1 absolutely, and the efflux of calcium from the ER through ryanodine receptor (RyR) channels and inositol triphosphate receptors (IP_3_R) channels in the ER membrane [27]. The released calcium is regenerative because it can be uptaken again by a Ca^2+^ -ATP_ase_ pump. Inhibition of the pump by thapsigargin can cause a reduction of the calcium store followed by an activation of the SOC, which will allow Ca^2+^ influx. The SOC-mediated capacitive Ca^2+^ influx is termed as store-operated Ca^2+^ entry (SOCE), which has been reported to play an important role in PAH PASMC proliferation and vascular remolding [4,24,26]. A few studies have suggested that TRPV1 may be involved in SOCE by functioning as Ca^2+^ release channels in the ER or participating in SOC formation [24,26].

[Ca^2+^]_i_ can cause PASMCs contraction by allowing actin to activate the myosin ATP_ase_ in a Ca^2+^ -calmodulin (CaM) -myosin light chain kinase (MLCK) -myosin regulatory light chain (RLC) pathway and promote PASMCs proliferation and migration by stimulating transcription factors (TFs), which constitute the important foundation of PAH primely [27–29]. There are many TFs contributing to PASMCs proliferation and migration, some of which are Ca^2+^ -dependent, such as nuclear factor of activated T lymphocytes (NFAT), cAMP response element-binding protein (CREB) and activator protein-1 (AP-1) [27,29].

### NFAT

NFAT, which were originally identified in T lymphocytes, are also expressed in PASMCs. When dephosphorylated by calcineurin (CaN), a Ca^2+^/CaM-dependent phosphatase, NFAT translocate from the cytoplasm into the nucleus to regulate gene transcription by associating with transcription coactivators and binding cognate DNA motifs at enhancer sites [27,30,31]. The NFAT family is composed of NFATc1 – NFATc4, which share the property of Ca^2+^/CaN -dependent nuclear translocation, and a fifth member, which is Ca^2+^-independent and distinctly different from the other four members [27]. The content of CaN and NFAT in PAH PASMCs is highly increased compared with that in the control group, and the inhibitor of CaN/NFAT can attenuate PASMCs proliferation [31]. Therefore, the Ca^2+^/CaN/NFAT pathway can become a central pathway for physical and chemical factors leading to PAH and is an important downstream signaling pathway of SOCE, which is the main candidate for PAH PASMCs contraction, proliferation, and migration in PAH by increasing [Ca^2+^]_i_ [31–33]. TRPV1, as a calcium influx channel, has also been reported to be followed by NFAT nuclear translocation, which may constitute the upstream pathway of diseases [23,34]. When translocating into the nucleus, NFAT associates with transcription coactivators, such as AP-1 and members of GATA family, to control gene expression [27]. As shown by song et al., NFATc2 can upregulate the expression of Bcl-2, an antiapoptotic protein, in PASMCs, after activated by stromal interaction molecule 2 (STIM2), a Ca^2+^ sensor in the ER that recruits Orail proteins to form SOC and induce SOCE. Therefore, they thought the STIM2/SOCE/Ca^2+^/CaN/NFATc2/Bal-2 pathway plays an essential role in the process of PAH by regulating PASMCs apoptosis [33]. NFAT has also been shown to regulate the expression of SM-myosin heavy chain, SM-α-actin, α1 integrin, and caldesmon genes. NFATc3 nuclear translocation has been shown to promote PASMCs hypertrophy and vascular remolding by enhancing the expression of SM-α-actin, a contractile protein required for SMCs development [35]. Despite these proliferation-promoting genes, NFAT can also regulate some inflammatory genes, such as interleukin-2 and thymic stromal lymphopoietin (TSLP), some of which may participate in the pathogenesis of PAH [30,34]. Jia et al. showed in their study that the increased [Ca^2+^]_i_ induced by TRPV1 could produce NFAT-upregulated TSLP transcription, which contributed to airway inflammation [34]. The downstream genes regulated by NFAT (the antiapoptotic gene Bcl-2, the hypertrophic gene SM-α-actin, the inflammatory gene IL-2 and TSLP) shown above are not reported to definitely be mediated by TRPV1/Ca^2+^ in PASMCs. As a calcium channel located in both the plasma membrane and the ER membrane, TRPV1 is very likely to mediate them and a significant number of undiscovered genes related to PAH through NFAT.

### CREB

CREB is a Ca^2+^-dependent TF that modulates gene expression by binding to the promoter region of target genes after translocating into the nucleus. This translocation necessitates its phosphorylation by Ca^2+^/CaM-dependent protein kinase II and Ca^2+^/CaM-dependent protein kinase IV [27,29]. The pCREB has been found to be significantly upregulated in IPAH PASMCs, accompanied by increased TRPV1 and [Ca^2+^]_i_. Therefore, the TRPV1/Ca^2+^/pCREB pathway may be involved in the pathogenesis of PAH [4,33]. In contrast, there are reports showing the downregulation of pCREB in PAH PASMCs and the protective role of CREB from proliferation and remolding by regulating phosphatase and tension homolog (PTEN), a tumor suppressor gene, and mitochondrial calcium uniporter (MCU), a mitochondrial calcium uptake protein that can decrease [Ca^2+^]_i_ [36,37]. These contradictory results may be due to the presence of three types of isoforms of CREB, which may produce different effects [38].

### c-jun and c-fos

c-jun and c-fos are immediate-early genes participating in the formation of AP-1, a family of proteins involved in cell proliferation, inflammation, and apoptosis by regulating the expression of target genes. The expression of c-jun and c-fos can be upregulated by calcium influx and is much higher in lung vessels from IPAH patients and modeled PAH rats than normal. Therefore, the Ca^2+^/c-jun and c-fos pathway may also be a signaling pathway for PASMCs proliferation [29,39]. Although there is no evidence that TRPV1 is responsible for this pathway, we cannot exclude the existence of the TRPV1/Ca^2+^/c-jun(c-fos) pathway in PAH; further investigation is needed.

**Figure 1.**
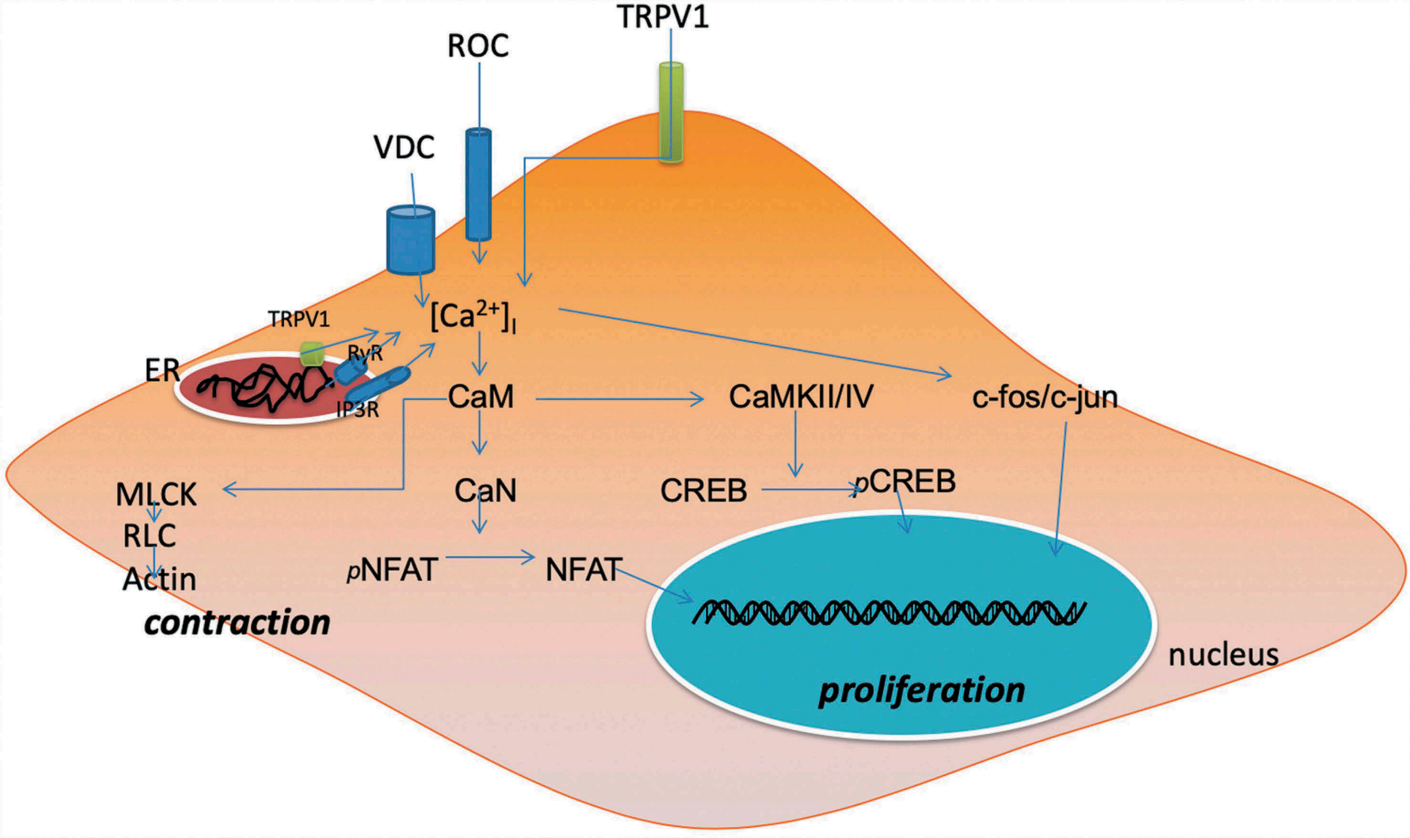
illustrates the contractive and proliferative role of increased [Ca^2+^]_i_ in PAH PASMC. The increased [Ca^2+^]_i_ coming from both Ca^2+^ influx and Ca^2+^ release can make PASMC contraction through the CaM/MLCK/RLC/actin pathway and aggravate PASMC proliferation by activating Ca^2+^ -dependent transcription factors, NFAT, CREB and c-fos/c-jun, which can translocate into nucleus to regulate the expression of target genes. ER: endoplasmic reticulum. TRPV1: transient receptor potential vanilloid subfamily member 1. VDC: Voltage-independent calcium channel. ROC: receptor-operated calcuim channel. RyR: ryanodine receptor channel. IP3R: inositol triphosphate receptor channel. CaM: calmodulin. CaN: calcineurin. MLCK: myosin light chain kinase. RLC: regulatory light chain. NFAT: nuclear factor of activated T cells. CREB: cAMP response element-binding protein. CaMKII/IV: CaM-dependent protein kinase II/IV.

Ultimately, the TRPV1/Ca^2+^/CaN/NFAT/Bal-2(TSLP, IL-2, α-actin) pathway, the TRPV1/Ca^2+^/CaN/CREB pathway and the TRPV1/Ca^2+^/c-jun(c-fos) pathway may be imprecise and sectional signaling pathways in the process of PAH. It seems that upregulation of TRPV1 expression in PASMCs can aggravate proliferation and migration via a Ca^2+^-dependent transcription pathway, so TRPV1 inhibition may be beneficial for PAH treatment. The role of Ca^2+^ in PASMC is illustrated in Figure 1. However, most of the described studies were in vitro experiments. In vivo, PASMCs are governed by nerves, and their phenotypes are more contracted and less proliferative under normal compared with in vitro conditions. Therefore, whether TRPV1 is a bad guy for PAH requires further study.

## TRPV1, inflammation and neuropeptides

An expanding body of knowledge has related inflammation in vascular cells to PAH pathogenesis. As Rabinovitch et al. showed in their review, in addition to increases in immune cell accumulation around perivascular tissue, there are abnormal elevations in circulating cytokines and chemokines during inflammation, some of which are closely related to PAH pathogenesis [40]. TRPV1 is well known to be expressed in terminals of sensory nerves and cause the release of neuropeptides, including proinflammatory CGRP and SP, and anti-inflammatory SST, which mediate local inflammation. The relationship between TRPV1 in nerves and inflammation in tissues seems to be bidirectional. TRPV1 can not only modulate inflammation by neuropeptides-induced events in the vascular system but also be affected by inflammatory factors secreted by immune cells and endothelial cells, such as IL-4, IL-13 and TNF-α [41–44]. Several researchers have demonstrated that TNF-α could exacerbate TRPV1-induced inflammation by upregulating the expression and sensitivity of TRPV1 after binding to its TRPV1-adjoined receptor in nerves [41–43].

Therefore, TRPV1 may participate in PAH pathogenesis by influencing the inflammatory process, although there have not been any specific studies on the inflammatory function of TRPV1 during the process of PAH. The relationship between TRPV1 and PAH may be closely associated with the neuropeptides released from the terminals of nerves. Because pretreatment with capsaicin, a specific activator of TRPV1, has been reported to alleviate PAH inflammation via depletion of proinflammatory neuropeptides in advance, and the p38 mitogen-activated protein kinase (MAPK) signaling pathway may participate in this process [45]. TRPV1-releasing neuropeptides exert their inflammatory function by binding to their specific subsets of receptors located in various cells including vascular endothelial cells, smooth muscle cells, immune cells and even neurons. Although these molecules are known as types of inflammation-related neuropeptides, their functions in vessels extend beyond mediating inflammation.

### CGRP

CGRP is a vasodilatory neuropeptide released from terminals of TRPV1-sensitive nerves, the receptors of which are a G protein-coupled receptor comprising a heterodimer containing a seven-transmembrane-domain ligand-binding protein, calcitonin-like receptor (CLR), and a single transmembrane protein, receptor activity-modifying protein 1 (RAMP1), which aids in the trafficking of CLR to the cell surface and generates pharmacological specificity. A third protein, CGRP receptor component protein (RCP), is a potential regulator of CLR/RAMP function in vivo and seems to be extraordinarily important for maintaining the function of CGRP [46–51].

CGRP has been described as a vasodilator for decades. It had been found to increase blood flow of various tissues and organs, including the skin, kidneys, brain, and coronary artery, and play a protective role in the progression of hypertension as a compensatory response to elevated blood flow [52]. It is well accepted that CGRP released from nerves can stimulate adenylate cyclase (AC), which will turn ATP into cAMP by binding to its receptor in VSMCs. The increased cAMP can change membrane voltage by activating protein kinase A (PKA)-induced K^+^ channels, which will inhibit VDCC followed by decreased [Ca^2+^]_i_-induced vasodilation. The receptor of CGRP also exists in endothelial cells, which can produce nitric oxide (NO) through the AC – cAMP – PKA pathway after activation. The diffusion of NO into adjacent VSMCs causes relaxation by activating guanylate cyclase [53]. As shown above, these processes are dependent on α subunits of GTP-binding regulatory proteins (Gα); however, there are reports demonstrating that βγ subunits of GTP-binding regulatory proteins (Gβγ) were responsible for these vasodilatory roles of CGRP via the kv7 family of potassium channels, although they failed to detail these roles. Despite this direct vasodilation, CGRP could inhibit vasoconstriction by preventing endothelin-1 from binding to its receptor in VSMCs. This anti-endothelinergic effect seems to be involved in Gβγ instead of Gα, as both the vasorelaxing and the anti-ET-1 effects of CGRP can be inhibited by Gβγ inhibitors [54,55].

As a vasodilator, CGRP can bring more blood flow to the area accompanied by increased amounts of immune cells and factors, participating in the inflammatory response in vivo [53]. Therefore, it has long been considered as a proinflammatory mediator, which can induce tissue hyperemia and edema, typically in migraine. However, it is too early to define CGRP as an inflammatory promotor. In some in vitro experiments, the general effects of CGRP seem to be anti-inflammatory. For example, in the early stages of bacterial pneumonia, CGRP released from TRPV1 nerve terminals could suppress neutrophil and γδ T cell responses [56]. In fungal osteoinflammation, CGRP was reported to inhibit cytokine production and tissue inflammation via suppression of NF-kB by transcriptional repressor jdp2, a member of the AP-1 gene, which is generally thought to be involved in TLR-mediated inflammation [57]. In a study of liver inflammation, it was found to reduce TNFα-production and protect the liver from injury inflammation [58]. When bound to its receptor on endothelial cells, CGRP also exerts anti-inflammatory effects by inhibiting the release of the chemokines CXCL8, CCL2, and CXCL1 via the NF-κB pathway [59]. According to the review by Assas et al., CGRP released from TRPV1 nerves could inhibit DCs maturation, antigen presentation, and motility; inhibit mast cell degranulation; mediate the differentiation of T cells Th2, which is associated with the production of anti-inflammatory cytokines, such as IL-4 [60].

Therefore, in the pathogenesis of PAH, whether CGRP acts as a proinflammatory one or anti-inflammatory mediator requires additional researches, and there have not been any studies focusing on the inflammatory role of TRPV1-induced CGRP in PAH. Previous studies seem to concentrate more on the antiproliferative role of CGRP in PAH. They demonstrated that CGRP could exert an antiproliferative role by increasing tumor-suppressive transcriptional factors, such as p53 and p27, which could inhibit PASMC proliferation by blocking the cell cycle [61,62].

### SP

SP is another important neuropeptide released from sensory terminals, regarded as proinflammatory factors together with CGRP. There are three types of neurokinin receptors, NK1, NK2, and NK3. SP shows the highest affinity to NK1, which is expressed in almost all endothelial cells [63,64]. SP can induce vasodilatory effects by interacting with endothelial NK1 receptors to phosphorylate endothelial nitric oxide synthase (eNOS), subsequently increasing NO-induced vasorelaxation, and by promoting the release of vasodilatory mediators, such as prostanoids and histamine, after interacting with receptors on white blood cells. In addition, it can degranulate mast cells, cause the accumulation of neutrophils, promote the adhesion of leukocytes, and activate immune cells [64,65]. All of these endow SP the proinflammatory characteristics. SP has been shown to stimulate the production of chemokines, such as TNF-α, IL-6 and MCP-1, and adhesion molecules, such as ICAM-1 and VCAM-1, by activating the transcription factor NF-kB, although the exact mechanisms of the association between SP and NF-kB differ and are vague in different cell types, and the extracellular signal-regulated kinase-1/2 (ERK1/2)-dependent activation of NF-kB downstream of PKC and the phosphoinositide 3-kinase (PI3K)-Akt pathway may be one signaling pathway responsible for SP-induced inflammation [66–68]. However, a recent study showed that SP could inhibit the macrophages release of TNFα and IL-6 by the retention of NF-kB in the cytoplasm, which indicates an anti-inflammatory effect of SP [69]. Actually, besides its vasodilatory and proinflammatory characteristics, SP also exerts vasoconstrictive effects by interacting with NK1 receptors on vascular smooth muscle cells [20]. Therefore, the vascular function of SP may be determined by the location of the binding receptors. It is worth mentioning that different concentrations of capsaicin have different effects on skeletal muscle arterioles, possibly because activation of TRPV1 in VSMCs can shrink vessels through Ca^2+^ signaling, whereas activation of TRPV1 in sensory nerves can dilate vessels through SP-endothelium interactions [70].

### SST

SST is an endogenous cyclic tetradecapeptide hormone that exists widely throughout the nervous system, gastrointestinal tract and pancreas and plays an important role in the modulation of hormones and neurotransmitter release, gastrointestinal functions, the cardiovascular system and the proliferation of tumor cells by binding to specific receptors in different cells. The receptor of SST is a G-protein-associated receptor that has five subtypes: sst_1–5_. On the basis of their binding profile towards synthetic SST analog, they can be divided into two subgroups: the SRIF1 group comprising sst_2_, sst_3_, and sst_5_, is able to bind to the octapeptide analog and is responsible for the antihormonal and antimitotic action, whereas the SRIF2 group, comprising sst_1_ and sst_4_, is able to bind to the heptapeptide analog and is responsible for the anti-inflammatory and antinociceptive action at both the vascular and cellular levels. In the nervous system, SST acts as a neuropeptide released from capsaicin-sensitive sensory nerve endings and exerts anti-inflammatory function by binding to receptors, generally sst_1_ and sst_4_, in nerve terminals, endothelial cells and immune cells. TT-232 and J-2156, the synthetic selective receptor agonists of sst_1_/sst_4_, have been reported to inhibit both the neurogenic and nonneurogenic vascular inflammatory response by suppressing CGRP/SP release and mast cell degranulation, respectively [71–73]. At the cellular level, as a G-protein-associated receptor, sst4 can exert an analgesic function by mediating the inhibition of AC activity and the downstream cAMP/PKA pathway, followed by inhibition of TRPV1 in neurons [74]. In peripheral cells, the anti-inflammatory role of SST may be related to the inhibition of NF-kB followed by decreased downstream expression of proinflammatory factors [15,75,76]. However, the TRPV1/SST/NF-kB signaling pathway requires further investigation.

**Figure 2.**
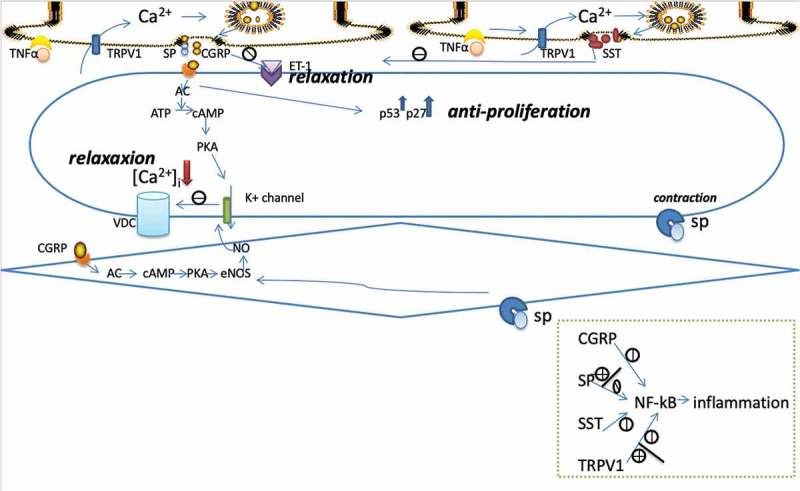
illustrates the vascular function of three neuropeptides released from capsaicin-sensitive nerves mainly. (1) Inflammatory factors, such as TNFα can active TRPV1 to release pro- and anti-inflammatory neuropeptides after binding to receptors adjoined to TRPV1. (2) combination of CGRP receptors on SMCs can decrease [Ca^2+^]i to cause vasorelaxation by activating AC/cAMP/PKA pathway, which opens K+ channels and inhibits calcium influx, and can inhibit SMC proliferation by upregulate P53 and P27. And if combined to endothelial cells, it can stimulate NO production, which diffuses into SMC to cause relaxation. Besides, CGRP can produce vasodilation indirectly by inhibiting ET-1 binding to its receptors. (3) SP combined to endothelial cells can dilate vessels via the eNOS/NO way. (4) SST can inhibit CGRP/SP release and TRPV1 activation. (5) TRPV1, CGRP, SP, and SST can mediate activities of inflammatory cells and productions of inflammatory factors by promoting or inhibiting expression of NF-kB. CGRP: calcitonin gene related peptide. SP: substance P. SST: somatostatin. TNFα: tumor necrosis factor α. ET-1 endothelin-1. TRPV1: transient receptor potential vanilloid subfamily member 1. VDC: Voltage-independent calcium channel. AC: adenylate cyclase. cAMP: cyclic adenosine monophosphate. PKA: protein kinase A. NO: nitric oxide. eNOS: endothelial nitric oxide synthase. NF-kB: nuclear factor-kappa B.

Activation of TRPV1 in sensory nerve terminals can release both proinflammatory and anti-inflammatory neuropeptides in vivo. The combined effect may determine the role of TRPV1 in the inflammatory response, especially in the pathogenesis of PAH, which is highly relevant to inflammation as shown above. Regrettably, there is a lack of direct research on the role of TRPV1 in PAH in terms of inflammation. However, in other inflammatory diseases, such as airway inflammation and arthritis, TRPV1 has been proven to exert beneficial effects via SST. Administration with SST analogs or TRPV1 agonists can alleviate synovial thickening, cell infiltration, cartilage destruction, and bone erosion in rats with artificial arthritis. In chronic arthritis, inflammatory factors, such as bradykinin, may selectively activate TRPV1 more in the SST-containing subpopulation of sensory nerves than in the CGRP/SP-containing subpopulation of sensory nerves. The released SST can inhibit the release of proinflammatory neuropeptides from nerves and inhibit systemic immune response processes, such as monocyte/macrophage functions, B lymphocyte immunoglobulin production, T lymphocyte proliferation, and cytokine production, by interacting with the appropriate receptors [14,77,78]. Therefore, we assume that TRPV1 may play an anti-inflammatory role by releasing SST after being activated by inflammatory mediators during PAH progression. The role of three neuropeptides in vessels is illustrated in Figure 2.

### TRPV1 in inflammatory cells

Apart from nerves, TRPV1 is also expressed in inflammatory cells to participate in inflammation directly. However, like TRPV1 in sensory nerves, the inflammatory role that TRPV1 plays in these cells is not consistent. Wang et al. demonstrated that activating TRPV1 in endothelial cells could exert an anti-inflammatory function characterized by a decrease in proinflammatory cytokine/chemokine production and adhesion molecule expression, as well as monocyte adhesion. This function may be due to the downregulation of NF-kB, a transcription factor modulating proinflammatory factor expression, by activation of TRPV1 via the Ca^2+^/PI3K/Akt/eNOS/NO pathway [79]. The anti-inflammatory role of TRPV1 is also found in dendritic cells (DCs), a kind of immune cell presenting antigens to T cells and producing different cytokines to participate in immune-inflammatory responses. Activation of TRPV1 could impede DCs maturation by downregulating toll-like receptor 4 and NF-kB [80]. However, in CD4 + T cells and lung epithelial cells, TRPV1 has been found to have a proinflammatory role by phosphorylating NF-kB [81,82]. Therefore, whether TRPV1 is a pro- or anti-inflammatory agent is controversial. There has even been research showing alternating effects of TRPV1 on inflammation. Wilhelmsen et al. believed that cannabinoids could change TRPV1 in human lung microvascular endothelial cells (HMVECs-lung) from being anti-inflammatory to being proinflammatory, as they found that inhibition of TRPV1 in HMVECs-lung could further reduce lipopolysaccharide-induced HMVEC-lung inflammation in the presence of cannabinoids but accelerate inflammation in the absence of cannabinoids [83].

Regardless of how complicated the role of TRPV1 is in inflammation, the effects of TRPV1 directly on inflammatory cells and indirectly on inflammatory cells through these three neuropeptides seem to all involve NF-kB, a transcription factor associated with proinflammatory factors, such as TNF-α, IL-6, MCP-1, ICAM-1, and VCAM-1. The proinflammatory effects are mediated by their upregulation of NF-kB, whereas the anti-inflammatory roles are due to their downregulation of NF-kB [59,66,75,79]. However, a large number of studies are necessary to detail the role of the TRPV1/NF-kB signaling pathway in inflammation, especially in the progression of PAH.

## Conclusion

In summary, TRPV1-induced increases in [Ca^2+^]_i_ in PASMCs can induce vascular contraction, proliferation and migration, which are enhancers of pulmonary hypertension. Besides, TRPV1 at sensory nerve termini can be upregulated by inflammatory cytokines to release the proinflammatory neuropeptides CGRP and SP, which can promote hypertension by disturbing the balance between vascular contraction and relaxation. However, TRPV1 can also release the anti-inflammatory neuropeptide, SST, which can inhibit not only the immune response but also the release of CGRP and SP, although the details of this process require further study. Furthermore, CGRP has been reported to prevent pulmonary arterial proliferation and inhibit chemokine release when bound to its receptors on smooth muscle cells and endothelial cells, respectively, which is beneficial for PAH. Thus, whether TRPV1 activation is an alleviator of PAH cannot currently be determined. Various inflammatory states, including PAH, are accompanied by increased TRPV1 expression, but whether TRPV1 is a cause of inflammation or a compensatory response after inflammation has not been determined based on related studies involving in vitro experiments. The human body as a whole performs many adjustments, and the ultimate effect of TRPV1 on PAH may be a combined result of Ca^2+^, neuropeptides and inflammation, and it may differ according to the concentration of agonists. Therefore, many more studies need to be performed to elucidate the relationship between TRPV1 and inflammation in terms of PAH. Such studies may enable us to clarify the pathogenesis of PAH and explore preventive and therapeutic strategies.
